# Supplementary material for: Identification of photoperiod-induced specific miRNAs in the adrenal glands of Sunite sheep (Ovis aries)
Source: Front Vet Sci. 2022 Jul 22;9:888207. doi: 10.3389/fvets.2022.888207 (PMC9354845; doi:10.3389/fvets.2022.888207)
Supplement: Supplementary file 1 [file Data_Sheet_1.ZIP › Supplementary file/miRDeep2 file.zip!/miRDeep2 file/Know_Structure/LP42A3/oar-mir-409.pdf]

miRBase precursor : oar-mir-409
Total read count : 25633
oar-miR-409-5p read count : 6071
oar-miR-409-3p read count : 19549
remaining reads : 13

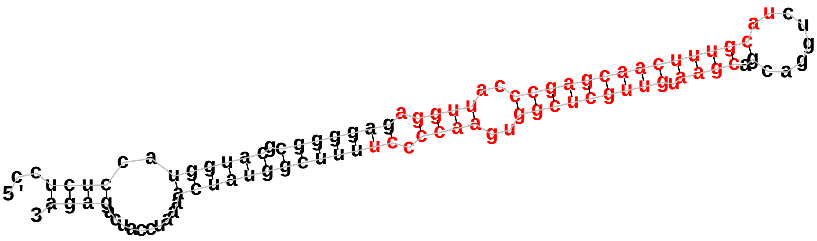

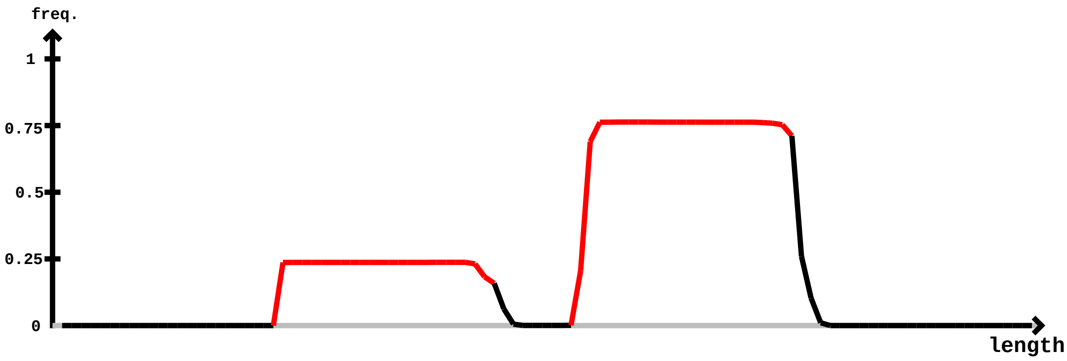


```
                                     oar-miR-409-5p
                                                                oar-miR-409-3p
5'- ccucuccaugguacgcggggagagguuacccgagcaacuuugcaucuggacgacgaauguugcucggugaaccccuuuucgguaucaaaauccaucagaga   -3'   exp
    .((((...((((((.(((((((.((((..(((((((((((((........).)))).)))))))))..)))).)))))))))))))........)))).   reads   mm   sample
    ......................agguuacccgagcaacuuu............................................................   4       0    seq
    ......................agguuacccgagcaacuuug...........................................................   153     0    seq
    ......................agguuacccgagcaacuuugc..........................................................   1243    0    seq
    ......................agguuacccgagcaacuuugca.........................................................   623     0    seq
    ......................agguuacccgagcaacuuugcau........................................................   2452    0    seq
    ......................agguuacccgagcaacuuugcauc.......................................................   1458    0    seq
    ......................agguuacccgagcaacuuugcaucu......................................................   129     0    seq
    .......................gguuacccgagcaacuuugc..........................................................   1       0    seq
    .......................gguuacccgagcaacuuugca.........................................................   1       0    seq
    .......................gguuacccgagcaacuuugcau........................................................   2       0    seq
    .......................gguuacccgagcaacuuugcauc.......................................................   1       0    seq
    ........................guuacccgagcaacuuugcau........................................................   2       0    seq
    ........................guuacccgagcaacuuugcauc.......................................................   2       0    seq
    ......................................uuugcaucuggacgacgaauguu........................................   10      0    seq
    ..........................................caucuggacgacgaauguugcu.....................................   1       0    seq
    ..........................................caucuggacgacgaauguugcucg...................................   2       0    seq
    ....................................................acgaauguugcucggugaa..............................   1       0    seq
    ....................................................acgaauguugcucggugaac.............................   4       0    seq
    ....................................................acgaauguugcucggugaacc............................   2       0    seq
    ....................................................acgaauguugcucggugaaccccu.........................   2       0    seq
    .....................................................cgaauguugcucggugaac.............................   30      0    seq
    .....................................................cgaauguugcucggugaacc............................   33      0    seq
    .....................................................cgaauguugcucggugaaccc...........................   63      0    seq
    .....................................................cgaauguugcucggugaacccc..........................   432     0    seq
    .....................................................cgaauguugcucggugaaccccu.........................   3241    0    seq
    .....................................................cgaauguugcucggugaaccccuu........................   1020    0    seq
    .....................................................cgaauguugcucggugaaccccuuu.......................   366     0    seq
    .....................................................cgaauguugcucggugaaccccuuuu......................   70      0    seq
    ......................................................gaauguugUucggugaac.............................   1       1    seq
    ......................................................gaauguugcucggugaac.............................   6       0    seq
    ......................................................gaauguugcucggugaacc............................   21      0    seq
    ......................................................gaauguugcucggugaaccc...........................   75      0    seq
    ......................................................gaauguugcucggugaacccc..........................   548     0    seq
    ......................................................gaauguugcucggugaaccccu.........................   7118    0    seq
```

oar-miR-409-5p

oar-miR-409-3p

ccucuccaugguacgcggggagagguuacccgagcaacuuugcaucuggacgacgaauguugcucggugaaccccuuuucgguaucaaaauccaucagaga

....gaauguugcucggugaaccccuu........................	2728	0	seq
....gaauguugcucggugaaccccuuu.......................	1756	0	seq
....gaauguugcucggugaaccccuuuu......................	149	0	seq
....gaauguugcucggugaaccccuuuuc.....................	3	0	seq
....aauguugcucggugaacc............................	1	0	seq
....aauguugcucggugaaccc...........................	10	0	seq
....aauguugcucggugaacccc..........................	84	0	seq
....aauguugcucggugaaccccu.........................	1219	0	seq
....aauguugcucggugaaccccuu........................	267	0	seq
....aauguugcucggugaaccccuuu.......................	238	0	seq
....aauguugcucggugaaccccuuuu......................	42	0	seq
....auguugcucggugaacccc..........................	1	0	seq
....auguugcucggugaaccccu.........................	2	0	seq
....auguugcucggugaaccccuuuu......................	3	0	seq
....uguugcucggugaaccccuu........................	1	0	seq
....uguugcucggugaaccccuuu.......................	9	0	seq
....uguugcucggugaaccccuuuu......................	3	0	seq
